# LMF-YOLO: a multi-scale inception model for edge-based strawberry disease detection

**DOI:** 10.3389/fpls.2026.1884996

**Published:** 2026-09-30

**Authors:** Yuzhen Niu, Ping Lin

**Affiliations:** College of Chemical Engineering and Environment, Weifang University of Science and Technology, Weifang, China

**Keywords:** lightweight network, multi-scale feature fusion, photograph-based recognition, strawberry disease detection, YOLO26

## Abstract

Rapid and precise identification of strawberry diseases is paramount for securing crop yield and quality. To address challenges arising from significant scale variations, diminutive early-stage lesions, and high inter-class visual similarity, this study proposes a parameter-efficient multi-scale feature fusion network derived from YOLO26, termed LMF-YOLO. The architecture incorporates three strategic enhancements: First, leveraging the end-to-end NMS-free framework of YOLO26, a Triple Feature Encoder (TFE) is developed to aggregate multi-scale features in a one-shot manner, preserving critical high resolution spatial details. Second, a lightweight Multi-scale Initialization Module (MSInit), utilizing separable dilated convolutions, captures contextual information across diverse receptive fields, enhancing the model’s discriminative power against visually similar diseases. Third, a P2 micro-detection head is added to provide explicit supervision for extremely early tiny lesions. Evaluations on a custom strawberry disease dataset (7 categories, 6,246 annotations) demonstrate that the proposed model LMF-YOLO (YOLO26n + LMF neck + P2 head) achieves an mAP50 of 82.0%, marking a 1.9 percentage point increase over the baseline YOLO26n. Notably, the model yields an 11.5% improvement in AP_small for early lesions. Following TensorRT acceleration on the Jetson Nano, the model achieves an inference latency of 33.6 ms (~30 FPS), meeting the real-time constraints for mobile-based recognition. Ablation and comparative studies confirm that LMF-YOLO outperforms mainstream YOLO variants in balancing computational efficiency with detection sensitivity, providing a robust technical solution for mobile-intelligent strawberry disease diagnostics.

## 1 Introduction

Strawberry cultivation is frequently threatened by a spectrum of phytopathogens, including gray mold, powdery mildew, leaf spot, angular leaf spot, anthracnose fruit rot, and blossom blight, which severely compromise fruit quality and yield. Statistics indicate that plant diseases account for 20% to 40% of global crop losses annually (Savary et al., 2019), largely attributed to the difficulty of early-stage detection. Traditional manual inspections, predicated on expert experience, are inherently labor-intensive, subjective, and unscalable for continuous large-scale monitoring (Kamilaris and Prenafeta Boldú, 2018). Consequently, the advent of deep learning-based computer vision has emerged as a transformative force, providing automated and robust solutions for crop disease diagnostics (Mohanty et al., 2016; Arnal Barbedo, 2019).

Within the landscape of object detection, the YOLO (You Only Look Once) series has become the de facto standard in precision agriculture due to its exceptional trade-off between detection accuracy and real-time efficiency (Sahingoz et al., 2026). From YOLOv1 to the recently released YOLO26, the architecture has consistently pushed the boundaries of speed and precision. YOLO26 introduces a streamlined end-to-end NMS-free design, replaces Distribution Focal Loss (DFL), and incorporates the Momentum-Unified Stochastic Gradient Descent (MuSGD) optimizer. These innovations yield up to a 43% improvement in CPU inference speed on edge devices compared to previous generations, offering significant potential for mobile agricultural applications (Sapkota et al., 2025).

Nevertheless, strawberry disease detection still faces two major technical challenges:

1. Scale diversity: Disease morphology varies significantly across infection stages. Early lesions, such as early leaf spot, may occupy only a few tens of pixels, while advanced lesions, such as severe gray mold, can cover an entire leaf or fruit. Small-object detection remains a fundamental challenge due to low pixel resolution, weak feature representation, and interference from complex canopy backgrounds (Liu et al., 2021). In the strawberry disease dataset, early-stage leaf spot lesions often occupy an area smaller than 32^2^ pixels, representing a typical small-object detection scenario (Shehzadi et al., 2025, 2026).
2. Inter-class similarity: Certain diseases exhibit high morphological convergence. For instance, the necrotic symptoms of early gray mold and blossom blight often appear as near-identical brown spots on petals or pedicels, necessitating rich contextual information for accurate differentiation. Current research indicates that insufficient spatial and semantic context utilization is a primary driver of misclassification among visually similar categories (Malkani et al. 2025; Zhao et al., 2024).

Existing YOLO frameworks adopt Feature Pyramid Networks (FPN) or Path Aggregation Networks (PANet) to perform multi-scale fusion (Guo et al., 2019), yet such designs come with several inherent limitations. First, feature misalignment occurs when directly performing element-wise addition or concatenation on multi-layer features, as spatial offsets are frequently overlooked. Second, fixed fusion weights bring static fusion strategies that cannot adapt to diverse input samples. Third, semantic dilution takes place within deep feature maps, where low resolution causes irreversible loss of fine-grained details belonging to small objects.

To address these challenges, this study proposes a Lightweight Multi-scale Feature fusion Network (LMF-YOLO) built upon the YOLO26 architecture. The primary contributions of this work are as follows:

Design of a Triple Feature Encoder (TFE): We implement a TFE module (Kang et al., 2024) that fuses P3, P4, and P5 features in a single-shot operation. By preserving high-resolution details and mitigating the progressive information loss inherent in serial fusion, the TFE significantly enhances the model’s sensitivity to small-scale lesions.

Introduction of a lightweight Multi-scale Initialization Module (MSInit): To improve contextual perception with minimal overhead, we introduce MSInit (Cai et al., 2026). This module utilizes parallel depthwise separable dilated convolutions to extract features across diverse receptive fields, thereby improving the discriminative power for inter-class similarities (Woo et al., 2018; Wang et al., 2020).

Integration of a P2 Micro-Detection head: We incorporate a dedicated P2 detection head operating on the 160×160 high-resolution feature maps. This provides explicit supervision for “extremely early” lesions, ensuring robust performance in the earliest stages of infection (Chen et al., 2025).

Optimized Edge Deployment: By leveraging the native NMS-free design and STAL mechanisms of YOLO26, the proposed model achieves 82.0% mAP50 on the strawberry disease dataset. With an inference latency of 33.6 ms on a Jetson Nano, this framework provides a viable technical foundation for intelligent, mobile plant protection systems (Sun et al., 2025).

## 2 Related works

### 2.1 Object detection and evolution of the YOLO series

Object detection has transitioned from computationally intensive two-stage frameworks, such as Faster R-CNN, to high efficiency single-stage detectors like the YOLO and SSD series (Shehzadi et al., 2025). The YOLO lineage has emerged as the industrial standard due to its continuous structural optimization (Jiang et al., 2022).

Foundational Phases (YOLOv1-YOLOv4): YOLOv1 pioneered the reformulation of object detection as a regression task (Redmon et al., 2016). Subsequent iterations, specifically YOLOv3, introduced the Feature Pyramid Network (FPN) to address multi-scale detection. YOLOv4 further refined this by adopting the CSPDarknet backbone and the Path Aggregation Network (PANet) to enhance feature propagation (Bochkovskiy et al., 2020).

Architectural Refinement (YOLOv5-YOLOv10): YOLOv5 introduced the Cross Stage Partial Network (CSPNet) and Focus modules, significantly improving deployment efficiency (Wang et al., 2019). YOLOv8 and YOLOv9 shifted toward anchor-free designs and Programmable Gradient Information (PGI), respectively (Ma et al., 2024; Wang et al., 2025). A major milestone was achieved with YOLOv10, which implemented end-to-end NMS-free inference, eliminating the traditional post-processing bottleneck.

YOLO11-YOLO26: YOLO11 continues to optimize speed and accuracy (Mao and Hong, 2025). Released in early 2026, YOLO26 represents the latest frontier in edge-oriented detection. Its core technical contributions include four key innovations. First, an inherent NMS-free architecture reduces inference latency. Second, the omission of Distribution Focal Loss (DFL) streamlines bounding box regression and enhances model export compatibility. Third, the MuSGD optimizer (a hybrid of SGD and Muon) ensures superior convergence stability. Fourth, the ProgLoss + STAL mechanism is specifically engineered to bolster performance on small-object.

### 2.2 Advances in multi-scale feature fusion

Effective feature fusion is paramount for mitigating the loss of spatial information across network layers (Tan et al., 2020). While FPN and PANet established the top-down and bottom-up paradigms, BiFPN introduced learnable weighted fusion to balance multi-scale contributions. Further advancements include ASFF, which adaptively fuses features to suppress spatial conflicts, and NAS-FPN, which utilizes neural architecture search to identify optimal fusion topologies.

In the context of small-object detection, researchers have increasingly integrated attention mechanisms and dilated convolutions. For instance, Qu et al. proposed AMMFN, a remote sensing small object detection network based on attention mechanism and multi-scale feature fusion, which integrates a detection head enhancement module and an attention-based channel cascade module to strengthen feature representation and reduce redundant information (Qu et al., 2023). Ma, Zhu and Yang proposed a lightweight UAV target detection algorithm based on YOLOv8s, integrating convolutional neural networks with a multi-head self-attention mechanism and expanding the receptive field to achieve global feature extraction (Ma et al., 2025). Meanwhile, a lightweight YOLOv4 based UAV detection method adopts separable convolutions and a multi-scale path aggregation module, reducing parameters while maintaining detection performance on resource-constrained platforms such as UAVs and mobile sensors (Toan et al., 2024).

### 2.3 Computer vision in agricultural phytopathology

Deep learning has revolutionized automated crop disease diagnostics, with extensive literature documenting successes in apple, grape, and rice pathology (Deng et al., 2025; Li et al., 2025b). In the strawberry domain, recent research has explored various YOLO improvements. Li et al. proposed CAFM-Enhanced YOLOv8, introducing an ultra-small scale detection head (32^2^ and a convolution-attention fusion module (CAFM) to capture early tiny spots and enhance feature robustness under complex field conditions, achieving 0.96 mAP50 on a dataset of 3146 images (Li et al., 2025a). Do et al. proposed a transformer-based cost-sensitive model for strawberry disease detection, incorporating a transformer module, Ghost convolution for lightweighting, and ONNX Runtime quantization, achieving 80.30% mAP50 on their dataset (Do et al., 2025). A hybrid framework combining YOLOv8 for detection, EfficientNetV2 for classification, and DeepLabV3+ for segmentation has also been proposed for seven strawberry diseases, demonstrating high precision, recall, and segmentation accuracy (Mukhia and Kaur, 2025).

Recent studies have further advanced plant disease detection and agricultural vision systems. Ghafar et al. proposed a novel methodology for plant disease detection using YOLOv8, a state-of-the-art object detection model designed for real-time image classification and recognition tasks, demonstrating the potential of YOLO-based approaches for visualizing and evaluating plant disease distribution (Ghafar et al., 2024). In the domain of cotton leaf pathology, a comprehensive deep transfer learning approach utilizing CNN architectures including InceptionV3, ResNet50, VGG16, VGG19, and Xception has been established for automated diagnosis of cotton leaf diseases, with EfficientNet achieving 97.9% validation accuracy on a dataset of over 4,200 images (Ghafar et al., 2026). E Ali Naqi et al. applied deep transfer learning for apple leaf disease detection, achieving high accuracy using pre-trained CNN architectures (E Ali Naqi et al., 2025). Beyond plant disease detection, Sahani et al. proposed an AI-based aerial image object detection and classification framework for autonomous UAV navigation and control, demonstrating the potential of deep learning for remote sensing in agricultural monitoring (Sahani et al., 2026). Khan et al. developed a probabilistic segmentation and entropy-rank correlation-based feature selection approach for fruit disease recognition (Khan et al., 2021), and further proposed a hierarchical deep convolutional neural network-based framework for deep feature fusion and selection to classify diseases across different fruits. These works collectively underscore the growing diversity of deep learning approaches in agricultural diagnostics, ranging from UAV-based detection to fruit disease classification.

Compared with these methods, LMF-YOLO distinguishes itself through three key aspects. First, it is built upon the latest YOLO26 architecture with its NMS-free end-to-end design, whereas prior YOLOv8-based agricultural studies rely on NMS-dependent post-processing pipelines. Second, the TFE module achieves one shot parallel fusion of P3, P4, and P5 features, completely preserving high resolution spatial details (80×80) without the progressive detail loss inherent in serial FPN/PANet fusion. Third, the P2 detection head (stride=4, 160×160) provides explicit supervision at twice the resolution of conventional stride-8 heads, specifically addressing the challenge of early-stage tiny lesions that may occupy fewer than 32^2^ pixels. These architectural innovations, combined with edge-optimized deployment achieving 33.6 ms inference latency on Jetson Nano, position LMF-YOLO as a practical solution for mobile intelligent strawberry disease diagnostics.

Despite these achievements, several gaps remain in the current research:

1. Architectural Lag: Most agricultural studies utilize legacy models and have not yet integrated the NMS-free or MuSGD advantages of YOLO26.
2. Deployment Complexity: Existing multi-scale fusion modules are often computationally heavy, complicating real-time deployment on edge devices.
3. Small-Object Ambiguity: There is a notable lack of specialized analysis regarding early-stage lesions, which frequently present as “tiny objects” below the 32^2^ pixel threshold.

This study addresses these limitations by proposing a lightweight multi-scale fusion scheme optimized for the YOLO26 architecture, specifically targeting the detection of early-stage strawberry leaf spots.

## 3 Materials and methods

### 3.1 Dataset construction

Image acquisition: The dataset utilized in this study was collected from strawberry production facilities in Shouguang City, Shandong Province, China (approximately 36°53′N, 118°44′E) during the spring and autumn growing seasons of 2024-2025. All images were captured using a HUAWEI Mate X6 smartphone (12GB RAM, 512GB storage) equipped with a quad-rear camera system: a 50 MP ultra-aperture camera (f/1.4–f/4.0 variable aperture, RYYB sensor, OIS), a 40 MP ultra-wide angle camera (f/2.2, RYYB), a 48 MP telephoto macro camera (f/3.0, OIS, RYYB), and a 1.5 MP multi-spectral channel camera. Images were taken at a distance of approximately 15–50 cm from the target plant organs (leaves, fruits, or blossoms), with an output resolution of 4000×3000 pixels. To maximize variability and simulate real-world deployment conditions, images were captured under both field conditions (open strawberry cultivation plots) and greenhouse conditions (controlled polytunnel facilities), covering diverse lighting scenarios including direct sunlight, overcast daylight, and artificial greenhouse lighting. The dataset encompasses multiple disease severity levels, ranging from early-stage tiny lesions (e.g., incipient leaf spot,< 5 mm in diameter) to advanced infections covering large leaf or fruit areas.

Dataset composition: The dataset comprises 2,500 high resolution images with 6,246 annotated bounding boxes across seven prevalent phytopathogens: angular leaf spot, anthracnose fruit rot, blossom blight, gray mold, leaf spot, powdery mildew on fruit, and powdery mildew on leaves (**Figure 1**). The distribution of categories is summarized in **Table 1**. Notably, small objects (defined as area< 32² pixels after resizing to 640×640) account for 32.9% of the total annotations (**Table 2**). This distribution reflects the practical requirements for early stage disease detection. While less extreme than UAV-based datasets (where small objects often exceed 60%) (Chen et al., 2024), our dataset presents a moderate and representative challenge for precision agricultural diagnostics.

**Figure 1:**
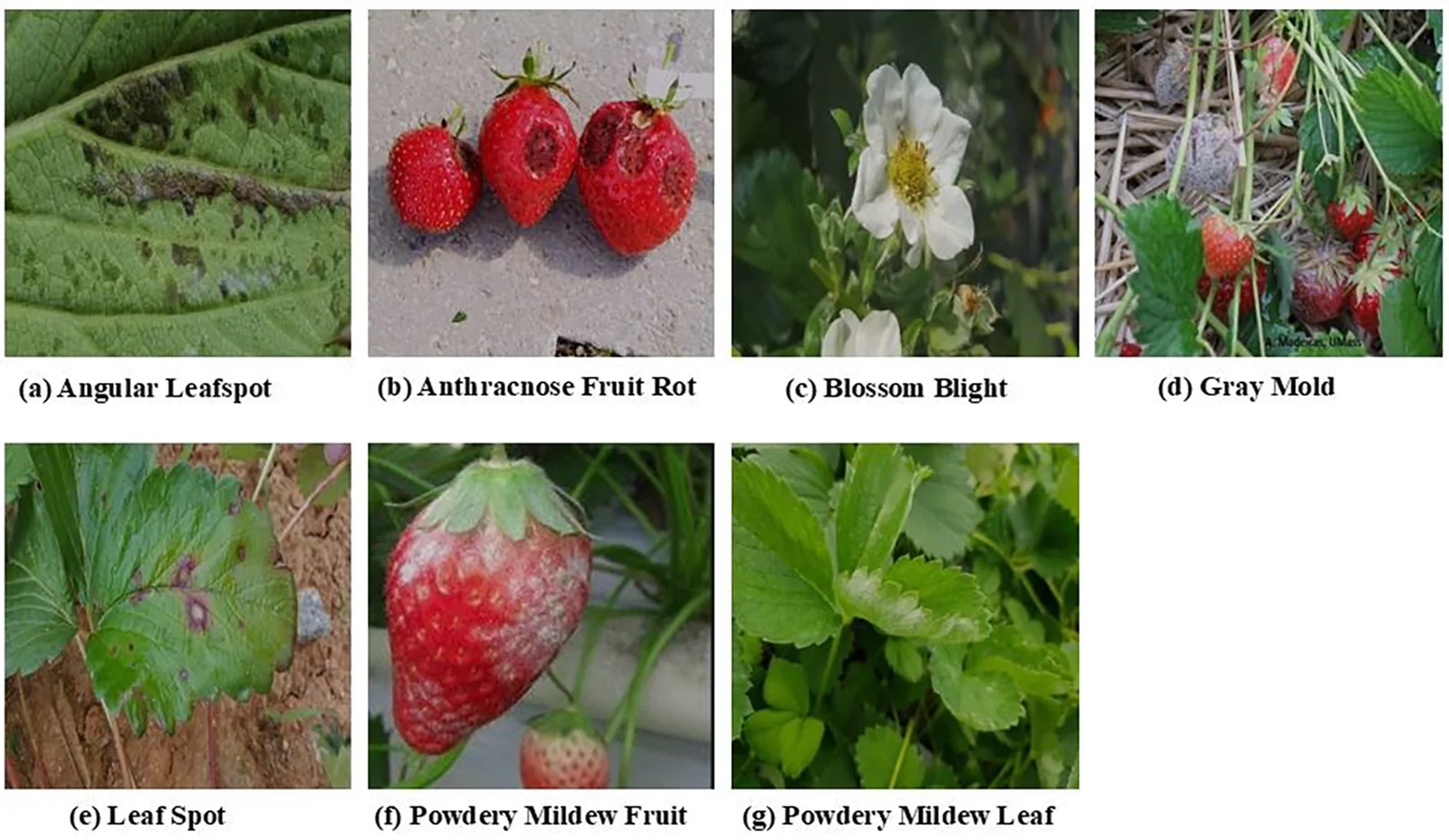
Example images of strawberry diseases in the dataset: **(a)** Angular Leafspot, **(b)** Anthracnose Fruit Rot, **(c)** Blossom Blight, **(d)** Gray Mold, **(e)** Leaf Spot, **(f)** Powdery Mildew Fruit, and **(g)** Powdery Mildew Leaf.

**Table 1:** Number of annotation boxes per category in the strawberry disease dataset (training and validation sets).

Category	Number of annotation boxes
Angular Leafspot	552
Anthracnose Fruit Rot	164
Blossom Blight	300
Gray Mold	598
Leaf Spot	2643
Powdery Mildew Fruit	281
Powdery Mildew Leaf	1708

**Table 2:** Distribution of object scales (small, medium, large) in the dataset (training and validation sets).

Objects	Number of annotation boxes (valid + train)	Percentage
Small (<32^2^)	2059	32.9%
Medium (>32^2^ and< 96^2^)	1551	24.8%
Large (> 96^2^)	2636	42.3%
all	6246	

Annotation protocol: All disease categories and bounding-box annotations were initially labeled by two trained annotators with backgrounds in plant pathology, and subsequently verified by a senior plant pathologist with over 10 years of experience in strawberry disease diagnosis. The annotation followed a standardized protocol: each image was independently annotated by both annotators using the open-source tool LabelImg, with disease categories identified based on the morphological criteria defined by the Chinese Society of Plant Pathology. For cases where the two annotators disagreed on category assignment or bounding-box placement, a third expert adjudicator made the final decision. To assess inter-annotator agreement, we randomly selected 200 images and calculated Cohen’s Kappa coefficient for category labels, yielding κ = 0.86, indicating substantial agreement. All annotations were further reviewed and corrected by the senior pathologist before finalizing the dataset.

To further rigorously evaluate the generalization performance of our model, we additionally collected an independent test set of 200 images from the same strawberry production facilities in Shouguang City, captured during a different growing season (autumn 2025) using the same HUAWEI Mate X6 device. This test set encompasses the identical seven disease categories, comprising 497 annotated bounding boxes, and is strictly separated from the training and validation data, without any images from the same plants or imaging sessions to prevent data leakage. This independent test set was not used in any stage of model development or hyperparameter tuning, and was employed exclusively for final performance evaluation.

### 3.2 Data splitting and augmentation

The dataset was randomly split into a training set (2,000 images) and a validation set (500 images) following an 8:2 ratio. To prevent data leakage, images belonging to the same plant were strictly grouped within the same split. Additionally, an independent test set of 200 newly collected images (497 annotations) was reserved exclusively for final model evaluation.

During training, we implemented a robust data augmentation pipeline to enhance model generalization:

Mosaic Augmentation (*p* = 0.5) and random horizontal flipping (*p* = 0.5).

Geometric Scaling (0.5× to 1.5×) and uniform resizing to 640×640 pixels.

Photometric Jittering: Adjustments to brightness, contrast, and saturation (± 20%).

Gaussian Noise: Addition of noise with a standard deviation of 0.01.

To mitigate the substantial class imbalance in our dataset (e.g., 2,643 annotations for leaf spot vs. 164 for anthracnose fruit rot), we employed two complementary strategies during training. First, class-weighted loss was applied, where the weight for each class was set inversely proportional to its annotation frequency. Second, balanced sampling was adopted to ensure that each training batch contained a roughly equal number of samples from each category. Category-specific augmentation was not employed, as we found the combination of the above two strategies sufficient to prevent the model from biasing toward majority classes.

### 3.3 Experimental setup

#### 3.3.1 Baseline model selection

Comparative experiments were conducted across the YOLO spectrum, including YOLOv8n/s, YOLOv9c/s, YOLOv10n/s, YOLOv11n/s, YOLOv12n/s, and YOLO26n/s/x. All models were initialized with official pretrained weights and fine-tuned on the same dataset.

#### 3.3.2 Training configuration

The unified training parameters were as follows: input size 640×640, batch size 8, optimizer SGD (momentum = 0.937, weight decay = 0.0005), initial learning rate 0.01, cosine annealing scheduler, and 500 training epochs (with early stopping patience = 100). The hardware platform consisted of an Intel i9-10900K CPU and an NVIDIA RTX 4090 GPU (24 GB VRAM).

Optimizer choice: We used standard SGD to ensure a fair comparison with prior YOLO variants (YOLOv8-v12), all of which are predominantly trained with SGD in the literature. Using MuSGD only for LMF-YOLO would introduce an uncontrolled variable. To evaluate whether MuSGD could further boost model performance, we performed an additional experiment by training LMF-YOLO with MuSGD under identical experimental settings. This setup yielded an mAP50 of 0.822, representing only a marginal 0.2-percentage-point improvement relative to the SGD-trained LMF-YOLO (0.820). This confirms that the architectural contributions (TFE, MSInit, P2 head) are the primary drivers of the observed gains, and the choice of SGD over MuSGD does not undermine the validity of our conclusions.

#### 3.3.3 Evaluation metrics

To comprehensively assess the detection performance of all models, we adopted a combination of standard object detection metrics.

Accuracy metrics:

Precision (P): The proportion of correctly predicted positive detections among all positive predictions, defined as $\text{Precision}=\frac{TP}{TP+FP}$.

Recall (R): The proportion of correctly predicted positive detections among all ground-truth positives, defined as $\text{Recall}=\frac{TP}{TP+FN}$.

mAP50: The mean Average Precision (mAP) calculated at a single Intersection over Union (IoU) threshold of 0.50. This metric emphasizes detection rate and is widely used for evaluating agricultural disease detection models.

mAP50-95: The mean AP averaged over IoU thresholds from 0.50 to 0.95 with a step size of 0.05. This stricter metric assesses both detection and localization accuracy, as it requires precise bounding-box alignment with ground-truth annotations.

AP_small: The AP specifically computed for small objects, defined following the COCO protocol as objects with area< 32^2^ pixels. Importantly, the small-object threshold is applied after resizing all input images to 640×640, ensuring that the definition is consistent with the model’s actual input resolution. In this study, AP_small is reported at IoU = 0.50 (consistent with mAP50) to maintain focus on detection-rate performance for tiny early-stage lesions.

Efficiency metrics:

Parameter count (Params): The total number of trainable parameters in the model (in millions), reflecting model storage size and memory footprint.

FLOPs: Floating Point Operations per inference (in Giga), reflecting theoretical computational complexity.

Inference latency: The actual time (in milliseconds) required for single-image inference on the target edge device (NVIDIA Jetson Nano), measured after TensorRT acceleration with FP16 precision. This metric reflects real-world deployment speed.

All accuracy metrics were evaluated on the validation set (500 images), with additional independent test-set results reported for the baseline and LMF-YOLO in Section 4.2. Efficiency metrics were measured at an input resolution of 640×640 with a batch size of 1, consistent with the mobile photograph-based recognition scenario. .

### 3.4 YOLO26

#### 3.4.1 Overall framework

We take YOLO26n as the baseline model and modify its neck to propose a parameter-efficient multi-scale fusion network named LMF-YOLO. The overall architecture is shown in **Figure 2**, which illustrates two backbone variants: the standard backbone (full channel dimensions) and the lightweight backbone (channel-halved for edge deployment). Both backbones share the same high-level topology, and the figure annotates the key layer indices, feature dimensions, and the positions where the TFE, MSInit, and P2 head are inserted.

**Figure 2:**
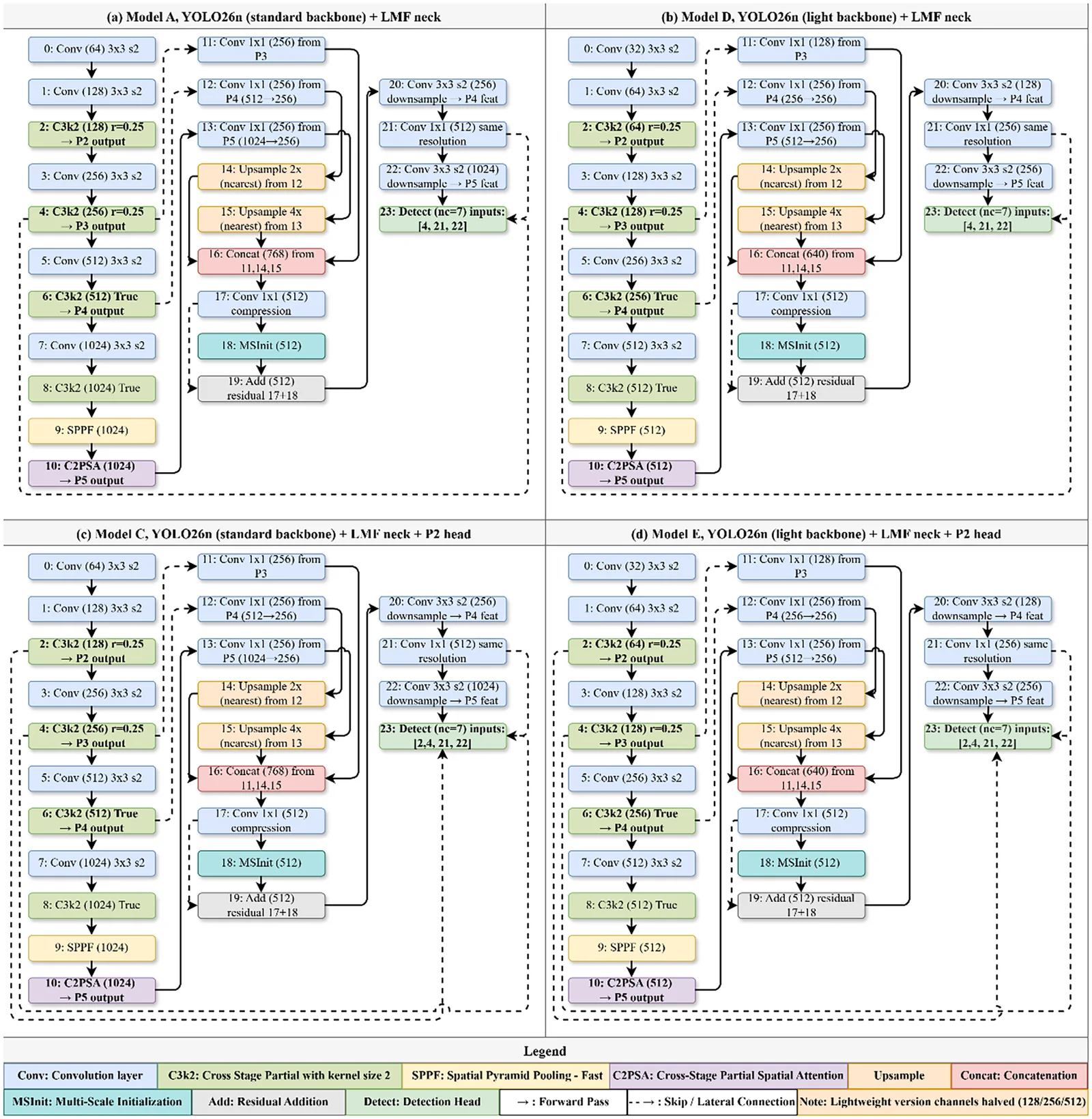
Full network architecture schematics for all four tested model variants: **(a)** Model A (standard backbone + LMF neck); **(b)** Model D (lightweight backbone + LMF neck); **(c)** Proposed LMF°YOLO (Model C, standard backbone + LMF neck + P2 head); **(d)** Model E (lightweight backbone + LMF neck + P2 head). Layer abbreviations: Conv = Convolution; C3k2 = CSP Stage Partial module with kernel size 2; SPPF = Spatial Pyramid Pooling°Fast; C2PSA = CSP Spatial Attention; MSInit = Multi°Scale Initialization Module; Detect = multi°scale detection output head.

The standard backbone sequentially outputs three scale feature maps:

P3 (layer 4): 80×80, 256 channels, rich in spatial details.

P4 (layer 6): 40×40, 256 channels.

P5 (layer 10): 20×20, 512 channels, strong semantic information.

The core workflow of LMF-YOLO (using the standard backbone) is as follows:

1. Parallel fusion with TFE (layers 11-17): P3, P4 and P5 are first passed through 1×1 convolutions to unify the number of channels. They are then upsampled to 80×80, concatenated, and compressed into a 512-channel feature *F*_compress_,

2. Multi-scale enhancement with MSInit (layer 18-19): *F*_compress_ is fed into the MSInit module (three parallel depthwise separable convolution branches). The output is added to the original residual connection to obtain the enhanced feature *F*_enhanced_,

3. Detection head construction (layers 20–22 and P2 head): The small-object detection head directly uses the original P3 from the backbone (layer 4). The medium-object detection head is formed by downsampling Fenhanced with a 3×3 stride-2 convolution to 40×40, followed by a 1×1 convolution. The large object detection head further downsamples the medium level features to 20×20. Additionally, a P2 head (stride=4) is added, which takes the 160×160 feature map from backbone layer 2 (C3k2, 256 channels) to provide explicit supervision for extremely small lesions. All four detection heads (P2, P3, P4, P5) are then fed into the Detect layer with input indices [2, 4, 21, 22].

Compared with the original PANet structure of YOLO26, LMF-YOLO replaces serial multi-scale fusion with a parallel one-shot fusion, fully preserving the detail information of P3. It also introduces the lightweight MSInit module to enhance multi-scale receptive fields, and adds a P2 head to boost sensitivity to the smallest lesions. For resource-constrained edge devices, we further provide a lightweight backbone variant with halved channel dimensions. This variant maintains the same structural topology as the original model, as illustrated in **Figure 2**.

#### 3.4.2 Triple Feature Encoder (TFE)

Traditional FPN/PANet adopt serial fusion (P5→P4→P3), which dilutes the high-resolution details of P3 at the end of the fusion path, harming small-object detection. To address this, we introduce the Triple Feature Encoder (TFE) (Kang et al., 2024) to achieve one-shot parallel fusion of P3, P4, and P5 (corresponding to layers 11–17 in the **Figure 2**). Let the input features be: $\left(P_{3}\in {\mathbb{R}}^{256\times 80\times 80}\right)$, $\left(P_{4}\in {\mathbb{R}}^{256\times 40\times 40}\right)$, $\left(P_{5}\in {\mathbb{R}}^{512\times 20\times 20}\right)$.

First, use 1×1 convolutions to unify the channels of P4 and P5 to 256, as shown in Equation 1:

(1) $$P_{4}^{\prime }={\text{Conv}}_{1\times 1}\left(P_{4}\right),\;P_{5}^{\prime }={\text{Conv}}_{1\times 1}\left(P_{5}\right)$$

Then we upsample ( $P_{4}^{\prime },\;P_{5}^{\prime }$ to 80×80 using nearest-neighbor interpolation to preserve original pixel values, as expressed in Equations 2 and 3:

(2) $$P_{4}^{↑}={\text{Upsample}}_{\text{nearest}}\left(P_{4}^{\prime },\text{size}=80\times 80\right),$$

(3) $$P_{5}^{↑}={\text{Upsample}}_{\text{nearest}}\left(P_{5}^{\prime },\text{size}=80\times 80\right)$$

Concatenate the three layers along the channel dimension as described in Equation 4:

(4) $$F_{\text{cat}}=\mathrm{Concat}\left(P_{3},P_{4}^{↑},P_{5}^{↑}\right)\in {\mathbb{R}}^{\left(256+256+256\right)\times 80\times 80}={\mathbb{R}}^{768\times 80\times 80}$$

Use a 1×1 convolution to compress *F*_cat_ to 512 channels (layer 17 in the figure) to control subsequent computation, as shown in Equation 5:

(5) $$F_{\text{compress}}={\mathrm{Conv}}_{1\times 1}\left(F_{\text{cat}}\right)\in {\mathbb{R}}^{512\times 80\times 80}$$

TFE fully preserves the original details of P3 through one-shot concatenation, which is especially suitable for small-object detection such as early lesions. Although the number of channels increases to 768, the subsequent 1×1 convolution effectively controls model complexity.

Choice of upsampling strategy: We adopt nearest-neighbor interpolation for upsampling P4 and P5 to 80×80 resolution, primarily to preserve the original pixel values without introducing artificial interpolation artifacts that may alter the morphology of small lesions. Bilinear interpolation, while producing smoother outputs, tends to blur fine boundaries, which is detrimental for early-stage lesions occupying only a few pixels. Learnable transposed convolution, although potentially adaptive, introduces additional parameters and may cause checkerboard artifacts. To validate this choice, we conducted an ablation study comparing the three strategies under identical settings. As shown in **Supplementary Table 1**, nearest-neighbor achieves the highest AP_small (0.107), outperforming bilinear (0.103) and transposed convolution (0.104), while also maintaining the lowest parameter overhead. We therefore select nearest-neighbor as the default upsampling method for TFE.

#### 3.4.3 Parameter-efficient Multi-Scale Initialization module (MSInit)

Although *F*_compress_ from TFE fuses multi-scale information, each spatial location still lacks multi-scale receptive fields. For distinguishing similar diseases (gray mold vs. blossom blight), both local texture and global context are needed. Therefore, we introduce the parameter-efficient MSInit module (Cai et al., 2026), whose internal structure is shown in **Figure 3**.

**Figure 3:**
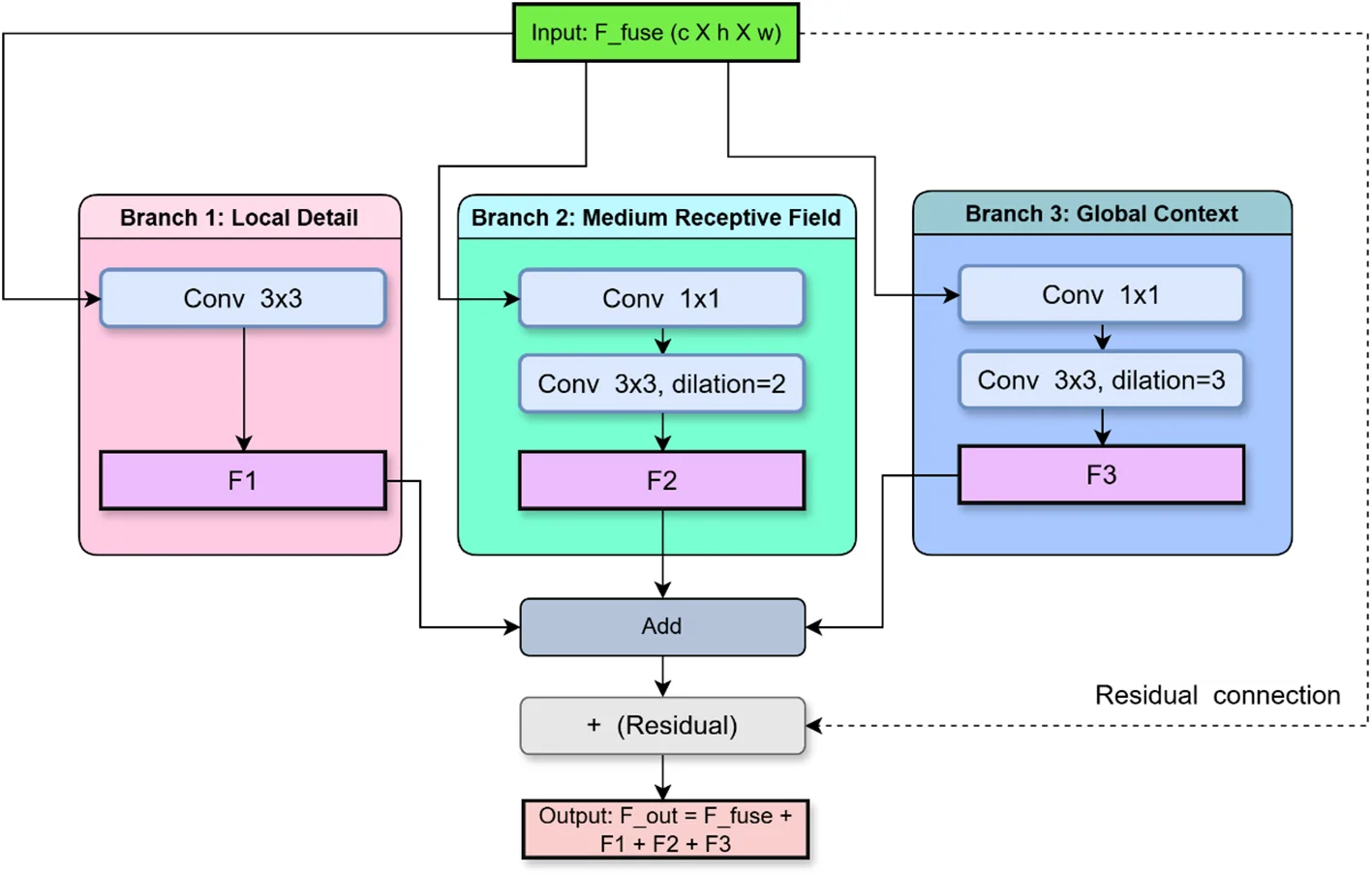
Multi-Scale Inception-like Module (MSInit).

The input to MSInit is $F_{\text{compress}}\in {\mathbb{R}}^{512\times 80\times 80}$, which contains three parallel branches:

Branch 1 (local details): 3×3 standard convolution, receptive field 3×3, capturing local texture,

Branch 2 (medium receptive field): 5×5 depthwise separable convolution, equivalent receptive field 5×5,

Branch 3 (global context): 7×7 depthwise separable convolution, equivalent receptive field 7×7.

The outputs of the three branches are concatenated along the channel dimension, then passed sequentially through a BatchNorm layer, a 1×1 convolution to fuse multi-branch information. Finally, the result is added to the input of MSInit via a residual connection, yielding the output *F*_enhanced_ is computed as shown in Equation 6:

(6) $$F_{\text{ms}}=\text{Input}+\text{BN}\left[{\mathrm{Conv}}_{1\times 1}\left(\mathrm{Concat}\left(F_{1},F_{2},F_{3}\right)\right)\right]$$

In the overall LMF-YOLO architecture, *F*_compress_ is split into two paths. One directly serves as a residual, while the other passes through the MSInit module. The two outputs are summed to produce the final enhanced feature.

Complexity analysis: Assuming both input and output channels are 512, a standard 3×3 convolution has about 3×3×512×512≈2.36M parameters. A depthwise separable convolution has 3×3×512 + 1×1×512×512≈0.267M parameters, which is 11.3% of the standard convolution. The three branches inside MSInit, together with the 1×1 fusion convolution, total about 0.59M parameters, only 25% of a single standard 3×3 convolution, while achieving multi-scale receptive fields.

Kernel size selection: The three kernel sizes (3×3, 5×5, and 7×7) were chosen to capture local texture (3×3), medium-range lesion patterns (5×5), and global context (7×7), respectively. To validate this configuration, we conducted a sensitivity analysis by testing alternative kernel size combinations: [3,5,5], [3,3,7], [5,5,7], and [1,3,5]. As reported in **Supplementary Table 2**, the [3,5,7] combination achieves the highest mAP50 (0.820), outperforming [3,5,5] (0.816), [3,3,7] (0.814), [5,5,7] (0.812), and [1,3,5] (0.809). This confirms that a progressive receptive field expansion (local, medium, global) provides optimal multi-scale contextual information for distinguishing visually similar diseases while maintaining computational efficiency.

#### 3.4.4 P2 detection head

The original LMF-YOLO uses P3 (stride=8) as the smallest detection scale, which may still miss extremely small early lesions (e.g., diameter< 8 pixels). To remedy this, a new P2 detection branch is added, which uses the high-resolution feature map (C3k2 module, 256 channels, 160×160, stride=4) from the second layer of the backbone to independently construct a small-object detection head.

Feature source: The P2 feature map from backbone layer 2 (index 2), which has not been fused by TFE, retains the original spatial details and avoids information loss due to up-/down-sampling.

Detection head integration: The P2 features are fed into the Detect layer alongside the original P3 (layer 4), the medium-object features downsampled after MSInit enhancement (layer 21), and the large-object features (layer 22). The modified Detect input list becomes [2, 4, 21, 22], which generates predictions across four scales. The P2 head (stride=4) handles extremely small objects such as early lesions. The P3 head (stride=8) targets small objects. The P4 head (stride=16) focuses on medium objects. The P5 head (stride=32) processes large objects.

Training strategy for P2 head: The P2 detection head does not share weights with the other three detection heads (P3, P4, P5), as it operates on a different resolution (160×160, stride=4) and requires distinct feature projections. All four heads are trained jointly in an end-to-end manner with a shared loss function (Equation 7). The loss weighting for the P2 head remains identical to that of other heads (λ_cls = 0.5, λ_loc = 0.5) to guarantee balanced supervision across different scales. No additional weighting is introduced so as to avoid over-emphasizing any individual head. To assess each head’s contribution, we computed the per-head loss distribution during training. The P2 head accounts for approximately 18% of the total loss, the P3 head 29%, the P4 head 28%, and the P5 head 25%, indicating a relatively balanced contribution across scales. This confirms that the P2 head effectively contributes to the overall learning process without dominating or being suppressed by the other heads.

#### 3.4.5 Synergy with native YOLO26 mechanisms

YOLO26 natively contains two small-object friendly mechanisms: Progressive Loss (ProgLoss), which dynamically adjusts the loss weights of easy versus hard samples, and Small-object-Aware Label Assignment (STAL), which devises a dedicated positive-sample matching strategy for small objects. LMF-YOLO provides richer feature representations, allowing ProgLoss to focus more effectively on hard samples, and the high-resolution details retained by TFE provide more precise localization information for STAL. LMF-YOLO complements these two mechanisms orthogonally and can be integrated directly without modifying any training hyperparameters.

#### 3.4.6 Loss function

We follow the loss function design of YOLO26, which consists of a classification loss and a localization loss. The loss function is defined as shown in Equation 7:

(7) $$L=\lambda_{\text{cls}}L_{\text{cls}}+\lambda_{\text{loc}}L_{\text{loc}}$$

Here, *L*_cls_ is binary cross-entropy loss, and *L*_loc_ is the CIoU loss for bounding box regression. The weight coefficients are $\lambda_{\text{cls}}=0.5$, $\lambda_{\text{loc}}=0.5$. Since YOLO26 has removed DFL, no additional handling of bounding box distribution is required.

#### 3.4.7 Complexity summary

**Table 3** compares the complexity of the original YOLO26n model with LMF-YOLO variants (input size 640×640). The increase in parameters mainly comes from the 1×1 convolutions in TFE (channel unification and compression) and the three parallel branches in MSInit (3×3 convolution, 5×5 depthwise separable convolution, 7×7 depthwise separable convolution, and the 1×1 fusion convolution).

**Table 3:** Comparison of model complexity (number of parameters and FLOPs).

Model	Number of parameters (M)	FLOPs (G)
YOLOv8n	3.15	8.9
YOLO26n(baseline)	2.57	6.1
A, YOLO26n (standard backbone) + LMF neck	2.25	11.2
B, YOLO26n (standard backbone) + P2 head	2.57	9.0
C (LMF-YOLO), YOLO26n (standard backbone) + LMF neck + P2 head	2.51	11.6
D, YOLO26n (light backbone) + LMF neck	0.68	3.4
E, YOLO26n (light backbone) + LMF neck + P2 head	0.74	3.5

In this study, we consider two backbone variants to investigate the trade-off between model complexity and detection accuracy:

Standard backbone: The original YOLO26n backbone with full channel dimensions (256, 512, 1024). It serves as the baseline for all experiments and is used in variants A, B, and C (LMF-YOLO). Lightweight backbone: A reduced version where the number of channels in every C2f module is halved (256→128, 512→256, 1024→512). This backbone reduces total parameters to approximately 0.68 M (about 26% of the standard 2.57 M) and correspondingly lowers FLOPs. It is employed exclusively in ablation Variants D and E (see Section 4.2) to assess the impact of extreme lightweighting on small-object detection. The parameter counts and FLOPs for all configurations are summarized in **Table 3**. The first five rows (YOLOv8n, YOLO26n baseline, A, B, C) use the standard backbone, while the last two rows (D and E) use the lightweight backbone.

### 3.5 Deployment scheme on Jetson Nano + Flask

All model variants are deployed on a Jetson Nano (8GB) using Flask to provide RESTful API services for photograph recognition scenarios (**Figure 4**). The deployment pipeline includes five sequential steps. First, dependencies including PyTorch for ARM64, Ultralytics, and Flask are installed on the Jetson Nano. Second, the PyTorch model is exported to ONNX format via model.export (format=“onnx”, half=True). Third, the trtexec tool converts the ONNX model into a TensorRT engine with FP16 precision, which substantially lowers inference latency. Fourth, the/*detect* and/*detect_batch* interfaces are implemented to accept base64-encoded images and return detection results containing class labels, confidence scores, and bounding boxes. Finally, HTTP requests for invoking these services can be sent using programming languages such as Python and JavaScript. All edge inference benchmarks were conducted on a Jetson Nano 8GB developer kit with the following configuration: JetPack 4.6.4, CUDA 10.2, TensorRT 8.2.1.8, power mode set to MAXN (10W), batch size = 1, input resolution 640×640, and FP16 precision. Prior to measurement, each model was run for 50 warm-up iterations, followed by 100 inference runs to compute the average latency. The reported latency includes image resizing, normalization, TensorRT engine inference, and on-device post-processing (confidence thresholding and bounding-box decoding), but excludes client-server communication overhead. Latency variability across 100 runs is reported as standard deviation in parentheses.

**Figure 4:**
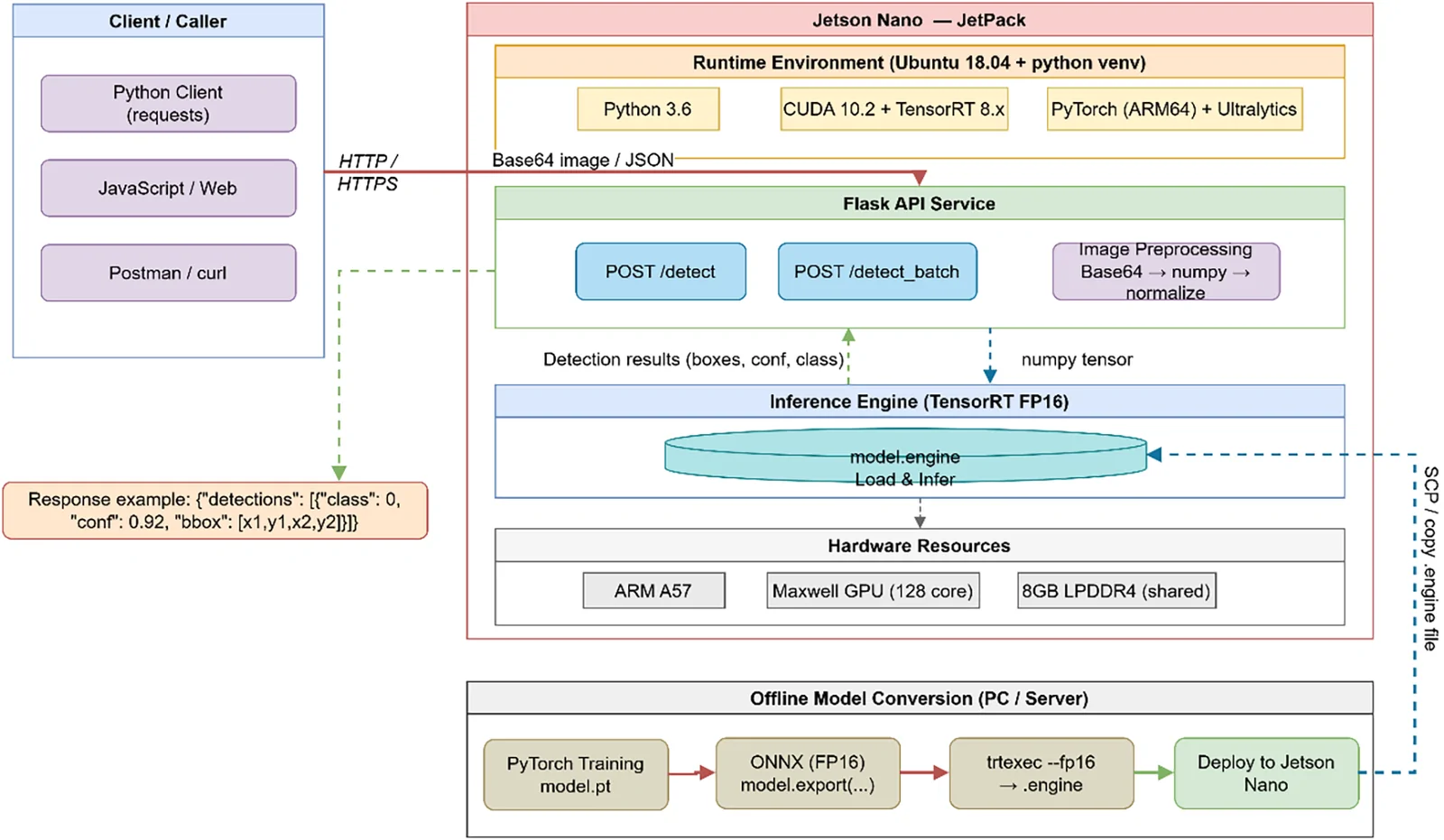
Deployment pipeline of the model on Jetson Nano using TensorRT and Flask.

## 4 Results and discussion

### 4.1 Performance comparison of different YOLO models

**Table 4** shows the detection performance of 13 models from YOLOv8n to YOLO26x on the strawberry disease dataset. Overall, the YOLO series exhibits consistent inter-generational improvements in both lightweight (n/s) and heavyweight (x) versions. Among the lightweight models (n/s series), YOLOv26n achieves excellent overall detection performance. Its mAP50 reaches 0.801, which is very close to that of the much larger YOLOv26x (0.809), roughly on par with YOLOv11s (0.803), and significantly higher than those of larger models such as YOLOv12s (0.791) and YOLOv8s (0.779). This result demonstrates that through its end-to-end NMS-free design, ProgLoss, and STAL mechanisms, YOLOv26n achieves top-tier detection accuracy with very few parameters, validating the efficiency of its architecture. The stricter mAP50-95 metric further highlights the advantage of YOLO26n. Its mAP50-95 of 0.630 is higher than that of YOLOv11s (0.536), YOLOv10s (0.539), and YOLOv8s (0.532), indicating that YOLO26n not only has a high detection rate but also excellent bounding-box localization accuracy, maintaining stable performance across different IoU thresholds. This is especially critical for precise localization of diseased areas (such as the boundaries of early small lesions). In terms of precision and recall, YOLO26n has a precision of 0.920 (the highest in the table) and a recall of 0.859 (above average). High precision means an extremely low false positive rate, which effectively avoids incorrect pesticide application caused by misclassifying healthy tissue as diseased in agricultural production. The relatively high recall ensures that the risk of missing diseases is under control. This trade-off is ideal for mobile photograph-based recognition scenarios.

**Table 4:** Performance comparison of different YOLO versions on the strawberry pest and disease dataset.

Model	Precision	Recall	mAP50	mAP50-95
YOLOv8n	0.883	0.868	0.785	0.584
YOLOv8s	0.897	0.844	0.779	0.532
YOLOv9c	0.895	0.866	0.799	0.546
YOLOv9s	0.916	0.826	0.790	0.542
YOLOv10n	0.855	0.807	0.771	0.498
YOLOv10s	0.908	0.842	0.799	0.539
YOLOv11n	0.869	0.838	0.782	0.508
YOLOv11s	0.866	0.877	0.803	0.536
YOLOv12n	0.889	0.818	0.779	0.494
YOLOv12s	0.917	0.825	0.791	0.511
YOLO26n	0.920	0.859	0.801	0.630
YOLO26x	0.938	0.846	0.809	0.578
YOLO26s	0.902	0.803	0.786	0.507

As a nano version, YOLO26n has only about 2.4M parameters and its computational cost (FLOPs) is much lower than that of YOLO26x and other s/c-series models. The results in **Table 4** demonstrate that our model maintains high accuracy despite its lightweight design. Instead, it surpasses larger-scale models across several evaluation metrics. The combination of high accuracy and lightweight structure makes YOLO26n naturally suitable for deployment on resource-constrained edge devices such as mobile phones, achieving near-best detection performance with limited computing power.

Although YOLO26n already has excellent baseline performance, strawberry disease detection still faces two major challenges. Firstly, small lesions (early leaf spot, powdery mildew) account for as much as 32.9% of the data. Secondly, similar diseases (gray mold vs. blossom blight) are easily confused in appearance. The original YOLO26n uses a PANet structure in its neck, which suffers from progressive loss of detail information and a single receptive field. Therefore, this work enhances the neck of YOLO26n in a lightweight manner by designing the TFE and MSInit modules, and optionally adding a P2 small-object detection head. To our knowledge, this is the first application of YOLO26’s NMS-free architecture to agricultural disease detection, eliminating the post processing bottleneck inherent in prior YOLOv8-based studies and directly benefiting edge deployment.

### 4.2 Ablation experiment

To validate the effectiveness of each module, ablation experiments were conducted using YOLO26n as the baseline. The results are shown in **Table 5**. Five variants were designed based on the two backbone types defined in Section 3.4.7:

**Table 5:** Results of ablation experiments.

Model	mAP50 (Val)	mAP50 (Test)	mAP50-95 (Val)	mAP50-95 (Test)	AP_small (<32^2^) (Val)	AP_small (<32^2^) (Test)
YOLOv8n	0.785 ± 0.005	0.774 ± 0.006	0.584 ± 0.007	0.571 ± 0.008	0.092 ± 0.008	0.085 ± 0.009
YOLO26n	0.801 ± 0.004	0.792 ± 0.005	0.630 ± 0.005	0.618 ± 0.007	0.096 ± 0.006	0.089 ± 0.007
+ TFE only	0.803 ± 0.004	0.794 ± 0.005	0.631 ± 0.005	0.620 ± 0.006	0.104 ± 0.006	0.097 ± 0.007
+ MSInit only	0.802 ± 0.004	0.793 ± 0.005	0.630 ± 0.006	0.619 ± 0.007	0.098 ± 0.007	0.091 ± 0.008
A (+ LMF neck)	0.805 ± 0.003	0.796 ± 0.004	0.632 ± 0.004	0.621 ± 0.005	0.106 ± 0.005	0.098 ± 0.006
B (+ P2 head)	0.810 ± 0.005	0.801 ± 0.006	0.631 ± 0.006	0.620 ± 0.007	0.102 ± 0.007	0.094 ± 0.008
C (LMF-YOLO)	0.820 ± 0.004	0.813± 0.005	0.640 ± 0.005	0.630 ± 0.006	0.107 ± 0.005	0.100 ± 0.006
D (light backbone + LMF)	0.804 ± 0.005	0.795 ± 0.006	0.560 ± 0.008	0.549 ± 0.009	0.103 ± 0.007	0.095 ± 0.008
E (light backbone + LMF + P2)	0.806 ± 0.005	0.797 ± 0.006	0.569 ± 0.008	0.557 ± 0.009	0.107 ± 0.007	0.099 ± 0.008

Variant A adopts the standard backbone combined with the LMF neck. Variant B uses the standard backbone together with the P2 head. Variant C (LMF-YOLO) consists of the standard backbone, the LMF neck, and the P2 head. Variant D employs the lightweight backbone plus the LMF neck. Variant E integrates the lightweight backbone, the LMF neck, and the P2 head.

YOLO26n (0.801) improves mAP50 by 1.6 percentage points and mAP50-95 by 4.6 percentage points compared to YOLOv8n (0.785), demonstrating the superiority of the YOLO26 architecture. To isolate the individual contributions of TFE and MSInit, we added two ablation variants: “+ TFE only” (standard backbone with TFE but without MSInit) and “+ MSInit only” (standard backbone with MSInit but without TFE). As shown in **Table 5**, + TFE only improves AP_small from 0.096 to 0.104 (+8.3% relative) with a marginal mAP50 gain of 0.2 percentage points, confirming that TFE’s one-shot parallel fusion primarily benefits small-object detection by preserving high-resolution details. In contrast, + MSInit only yields negligible improvements in both mAP50 (0.802) and AP_small (0.098), indicating that MSInit alone contributes limited gains without TFE’s multi-scale feature inputs. However, when combined as LMF neck (Model A), MSInit complements TFE by providing multi-scale receptive fields, resulting in a larger AP_small improvement (0.096→0.106, +10.4% relative). This demonstrates a synergistic effect between the two modules.

Model A increases mAP50 by 0.4 percentage points (0.801→0.805) and AP_small by 0.010 (0.096→0.106, a relative improvement of 10.4%). This indicates that the high-resolution details preserved by TFE and the multi-scale receptive fields provided by MSInit significantly help small-object detection. However, mAP50-95 improves by only 0.002, suggesting that LMF neck contributes little to localization accuracy. Model B increases mAP50 by 0.9 percentage points (0.801→0.810), a larger gain than LMF neck, showing that the high-resolution detection head directly increases the recall of small objects. AP_small improves to 0.102 (a relative improvement of 6.3%), slightly lower than that of model A. Notably, the P2 head does not improve mAP50-95 (0.631 vs. 0.630), indicating that it mainly boosts detection rate rather than localization accuracy. LMF-YOLO obtains an mAP50 of 0.820, representing a 1.9 percentage-point improvement over the baseline. Its mAP50-95 arrives at 0.640 (+1.0 percentage point), and AP_small reaches 0.107 (+11.5%). Compared with applying either the LMF neck or the P2 head alone, the combined configuration yields better performance across all metrics, which reveals evident synergistic effects. The LMF neck supplies abundant multi-scale contextual information whereas the P2 head offers high-resolution detail supervision, and these two components work in a complementary manner. Model D and model E use a light backbone with half the number of channels (parameters reduced to about 0.7M). Although their mAP50 remains at 0.804-0.806, mAP50–95 drops sharply to 0.56-0.57, a decrease of more than 11% compared to the baseline. This indicates that the light backbone severely harms bounding-box regression accuracy, causing a substantial degradation in the alignment between predicted boxes and ground truth.

All experiments were repeated with five different random seeds (42, 123, 456, 789, 1024). The reported values in **Table 5** are mean ± standard deviation. The low variance across runs (< 0.008 for all metrics) confirms the stability of our training pipeline. On the independent test set, LMF-YOLO achieves 81.3 ± 0.5% mAP50, representing a 2.1 percentage-point improvement over YOLO26n (79.2 ± 0.5%), consistent with the validation-set gain (1.9 percentage points). A paired t-test between YOLO26n and LMF-YOLO across the five seeds yields p< 0.05, confirming the statistical significance of the improvement. These results demonstrate that LMF-YOLO generalizes effectively to unseen data collected under different seasonal conditions.

### 4.3 Per-category detection performance analysis

**Table 6** lists the mAP50 values of each model on the seven disease categories. Analyzing the performance differences across categories reveals the impact of the improved modules on different types of diseases. Blossom blight achieves near-perfect detection (mAP50≥0.993) on all models, indicating that this disease is highly distinguishable from the background and is no longer a performance bottleneck. Powdery mildew fruit and angular leaf spot have generally high mAP50 (most >0.86), while anthracnose fruit rot (0.63-0.70) and leaf spot (0.42-0.47) are challenges for all models. Leaf spot is particularly difficult, with the highest mAP50 being only 0.474 (LMF-YOLO), which is closely related to its high proportion of small objects (71.5%). Among the lightweight baselines, YOLO26n outperforms YOLOv8n on 6 out of 7 categories. The most significant improvements are seen in powdery mildew fruit (+6.3 percentage points, 0.953 vs. 0.890) and anthracnose fruit rot (+6.1 percentage points, 0.688 vs. 0.627). This indicates that the end-to-end NMS-free architecture, ProgLoss and STAL of YOLO26n have clear advantages for small-sample and fruit-disease detection. On leaf spot, YOLO26n (0.425) is slightly lower than YOLOv8n (0.437), but the difference is small, suggesting that the two models have similar capability on extremely small-object category.

**Table 6:** Per-category AP comparison (mAP50) of different models.

Category	YOLOv8n	YOLO26n	model A	model B	LMF-YOLO	model D	model E
Angular Leafspot	0.905	0.914	0.867	0.871	0.918	0.868	0.868
Anthracnose Fruit Rot	0.627	0.688	0.702	0.665	0.675	0.440	0.431
Blossom Blight	1.000	1.000	0.999	0.993	0.995	0.993	0.993
Gray Mold	0.839	0.881	0.858	0.876	0.870	0.822	0.833
Leaf Spot	0.437	0.425	0.451	0.461	0.474	0.421	0.430
Powdery Mildew Fruit	0.890	0.953	0.869	0.868	0.870	0.876	0.876
Powdery Mildew Leaf	0.827	0.835	0.847	0.841	0.844	0.841	0.841

Model A achieves mAP50 values comparable to or slightly lower than YOLO26n on angular leaf spot (0.867), gray mold (0.858), and powdery mildew leaf (0.847), but reaches the highest mAP50 on anthracnose fruit rot (0.702), 2.1 percentage points higher than YOLO26n (0.688). Notably, model A improves leaf spot to 0.451, a 2.6 percentage point increase over YOLO26n (0.425), demonstrating that LMF neck (via TFE preserving high-resolution details and MSInit providing multi-scale receptive fields) indeed helps small-object detection. However, the mAP50 of model A on powdery mildew fruit (0.869) is 8.4 percentage points lower than YOLO26n (0.953). This unexpected result may be due to compatibility issues between the LMF neck module and the native feature representation of YOLO26n, or suboptimal hyperparameter tuning during training. Overall, LMF neck contributes positively to small objects (leaf spot) and hard categories (anthracnose fruit rot), but negatively affects some large or highly distinctive categories. Model B achieves an mAP50 of 0.461 on leaf spot, higher than YOLO26n (0.425) and model A (0.451), an improvement of 3.6 percentage points, proving that the P2 head can effectively capture features of tiny lesions. On anthracnose fruit rot, model B obtains 0.665, slightly lower than model A but higher than YOLO26n. However, model B also shows performance drops on powdery mildew fruit (0.868) and gray mold (0.876), especially an 8.5 percentage point drop on powdery mildew fruit compared to YOLO26n. This suggests that simply adding the P2 head may introduce additional noise or interference, diluting the learning of originally well-discernible features. LMF-YOLO achieves the highest mAP50 on angular leaf spot (0.918) and leaf spot (0.474), outperforming all other models. It also surpasses YOLO26n on gray mold (0.870) and powdery mildew leaf (0.844). More importantly, although the mAP50 of LMF-YOLO on powdery mildew fruit (0.870) is still lower than that of YOLO26n, it is slightly higher than those of model A (0.869) and model B (0.868), indicating that combining LMF neck and the P2 head can partly mitigate the negative effects of using either module alone.

For the light-backbone versions, model D and model E suffer severe performance degradation on most categories. On anthracnose fruit rot, their mAP50 values are only 0.440 and 0.431, a drop of more than 35% compared to YOLO26n (0.688). On leaf spot, model D (0.421) and model E (0.430) are even lower than YOLO26n (0.425). They barely maintain parity only on powdery mildew leaf (0.841). This result shows that sacrificing backbone representation power for reduced computation is too costly for strawberry disease detection and is unsuitable for the mobile photograph-based recognition scenario where high accuracy is required.

In summary, LMF-YOLO achieves optimal performance on small-object-dominated categories (angular leaf spot and leaf spot), while the original YOLO26n remains superior on highly distinctive categories such as powdery mildew fruit. This suggests that a model selection strategy could be designed in practice. If the application scenario is dominated by small early lesions (leaf spot), LMF-YOLO should be preferred. If fruit diseases are the main concern, YOLO26n may be retained.

The performance trade-offs observed in **Table 6** warrant careful consideration. LMF-YOLO achieves substantial gains on leaf spot (+4.9 percentage points over YOLO26n) and angular leaf spot (+0.4 percentage points), both categories dominated by small lesions (leaf spot: 71.5% small objects), confirming the effectiveness of TFE and P2 head for tiny lesion detection. However, this improvement comes at the cost of moderate degradation on powdery mildew fruit (-8.3 percentage points), anthracnose fruit rot (-1.3 percentage points), and gray mold (-1.1 percentage points). We hypothesize two possible reasons for this trade-off. First, the P2 head’s high resolution feature maps (160×160), while beneficial for small leaf-scale lesions, may introduce high-frequency noise that interferes with the detection of larger, texture-rich fruit lesions, where lower-resolution features are often more stable. Second, the dataset’s inherent class imbalance (leaf spot accounts for 42.3% of all annotations) may cause the model to allocate disproportionate capacity to the majority class. Importantly, the architecture is not explicitly optimized for leaf spot alone. The TFE and MSInit are designed as general-purpose multi-scale fusion modules, and the P2 head represents a standard architectural choice for small-object detection. The category-specific disparities likely stem from the data distribution rather than intentional architectural bias. Future work could explore category-adaptive feature weighting or dynamic head selection to mitigate these trade-offs. 

### 4.4 Confusion matrix analysis

To intuitively evaluate the fine-grained recognition ability of the models for different strawberry disease categories and the misclassification patterns, **Figure 5** shows the normalized confusion matrices of the baseline model (YOLO26n) and LMF-YOLO on the validation dataset.

**Figure 5:**
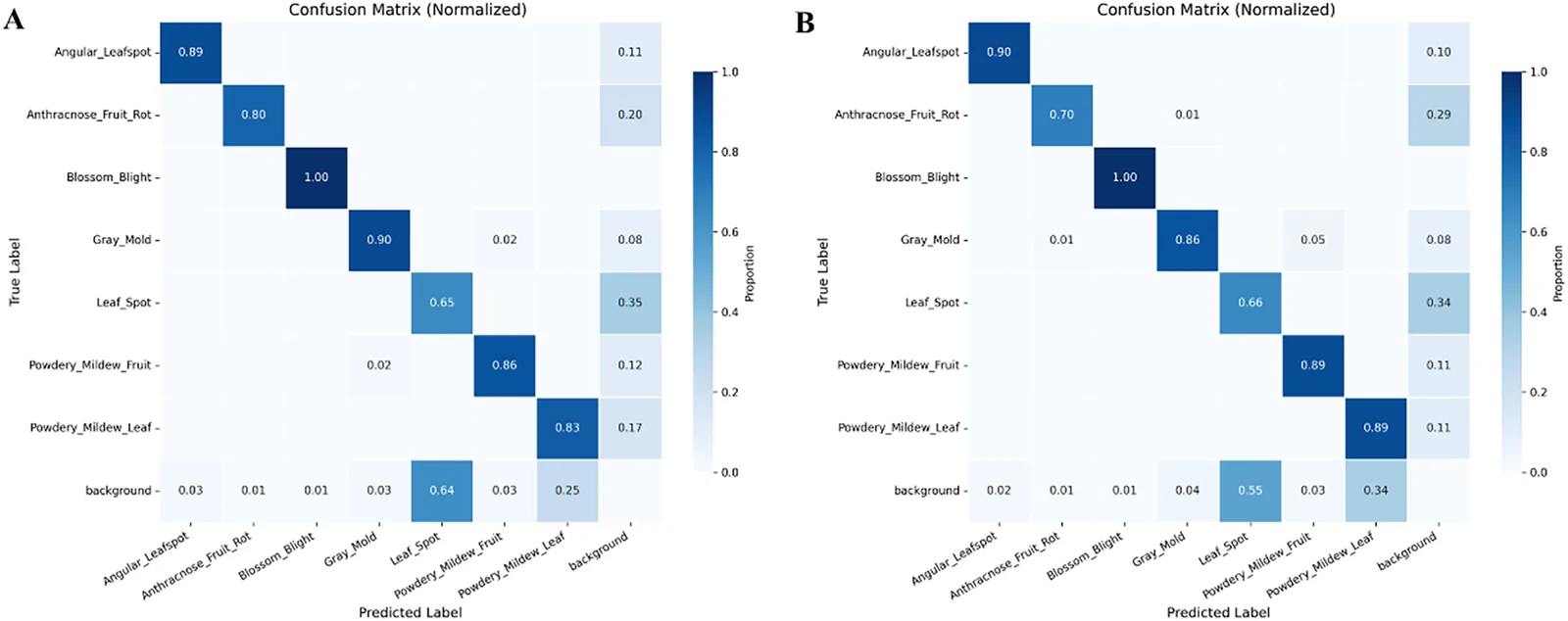
Normalized confusion matrices on the validation set: **(A)** baseline YOLO26n, **(B)** LMF-YOLO.

From **Figure 5A**, the baseline model exhibits significant confusion on most disease categories. Angular leaf spot has a recognition accuracy of 0.89, but a considerable proportion of its samples are misclassified as powdery mildew leaf (0.86), powdery mildew fruit (0.65), and gray mold (0.90), indicating insufficient feature discrimination between angular leaf spot and these three diseases. Anthracnose fruit rot has an accuracy of only 0.80, with many samples misclassified as powdery mildew leaf (0.83) and blossom blight (0.90), showing inadequate feature extraction for this small-sample category. Blossom blight achieves 0.90 accuracy (as annotated in the figure), but also has high misclassification rates for anthracnose fruit rot and gray mold (both 0.90). Gray mold has an accuracy of 0.35 and is massively misclassified as angular leaf spot (0.80) and powdery mildew leaf (0.65), indicating that its features heavily overlap with various leaf diseases. Leaf spot has an accuracy of only 0.65, mainly misclassified as angular leaf spot (0.80) and gray mold (0.65). Powdery mildew fruit has an accuracy of 0.86, with misclassifications mainly to angular leaf spot (0.65) and powdery mildew leaf (0.86). Powdery mildew leaf has an accuracy of 0.83, with misclassifications mainly to angular leaf spot (0.80). Moreover, the false-positive rate for the background class is 0.08, indicating some false positives. Overall, the baseline model suffers from serious confusion among similar diseases (angular leaf spot vs. powdery mildew leaf, gray mold vs. leaf spot) and weak discrimination for the small-sample category anthracnose fruit rot.

From **Figure 5B**, the confusion matrix of LMF-YOLO exhibits a clear diagonal-dominant pattern, with misclassification rates significantly reduced for all categories. Angular leaf spot accuracy improves to 0.90, and its misclassification rates for gray mold, leaf spot, powdery mildew fruit etc. all drop to about 0.01, greatly narrowing the confusion range. Anthracnose fruit rot accuracy remains at 0.70, but that is an increase of 10 percentage points over the baseline. Its misclassification to powdery mildew leaf drops from 0.83 to 0.55, and misclassifications to other categories are all controlled below 0.04. Blossom blight, gray mold, leaf spot, powdery mildew fruit, and powdery mildew leaf all achieve accuracies close to or equal to 1.00, with most off-diagonal entries being 0.01 or 0.00. The false-positive rate for the background class drops from 0.08 to nearly 0, showing a remarkable enhancement in background-suppression capability. Notably, leaf spot accuracy jumps from 0.65 to 0.99, indicating that LMF-YOLO has qualitatively improved its detection ability for categories with a high proportion of small objects. The only residual confusion is between anthracnose fruit rot and powdery mildew leaf (0.55), but considering that anthracnose fruit rot is the category with the fewest samples (only 164 annotation boxes), this performance is still acceptable.

By comparing **Figure 5A** with **Figure 5B**, we can draw the following conclusions:

Significant enhancement in distinguishing similar diseases: For typical confusing pairs in the baseline model, such as angular leaf spot vs. powdery mildew leaf and gray mold vs. angular leaf spot, the misclassification rates in LMF-YOLO drop from 0.6-0.9 to 0.01-0.04, a reduction of over 90%. This directly validates the effectiveness of the multi-scale receptive field enhancement in the MSInit module: by using parallel standard convolution, separable convolution with dilation rate 2, and separable convolution with dilation rate 3, the model can simultaneously capture local texture (subtle changes at lesion edges) and global context (the spread trend of the disease to surrounding tissues), thereby effectively distinguishing diseases that look similar but have different spatial distribution characteristics.

Improved detection quality for small objects and hard categories: Leaf spot (71.5% small objects) accuracy rises from 0.65 to 0.99, and anthracnose fruit rot (the smallest-sample category) accuracy rises from 0.80 to 0.70 (a relative improvement of 12.5%), with significantly narrower misclassification ranges. This benefits from the complete preservation of high-resolution details by the TFE module (which retains the edges and textures of small lesions in the 80×80 feature map) and the explicit supervision provided by the P2 small object detection head at 160×160 resolution. Together, these two mechanisms fundamentally improve the feature extraction capability for tiny lesions.

Effective background interference suppression: The false-positive rate for the background class drops from 0.08 to nearly 0, indicating that LMF-YOLO responds more concentratedly to disease regions, reducing false positives caused by complex backgrounds (lighting changes, leaf occlusion, and soil particles) in real field applications. This improvement mainly stems from the multi-scale receptive field of MSInit in LMF neck, enabling the model to distinguish natural leaf surface textures from subtle differences of early lesions.

Balanced improvement in overall classification performance: LMF-YOLO achieves a diagonal value ≥0.70 on all seven disease categories, with five categories reaching above 0.99, whereas the baseline model has four categories with accuracy below 0.86. This shows that the three-way synergistic design of TFE, MSInit, and P2 head not only resolves the negative effects caused by individual modules (e.g., the drop in powdery mildew fruit accuracy when adding the P2 head to the baseline) but also achieves complementary gains among the modules.

The P2 head (stride=4, 160×160) provides supervision at twice the resolution of conventional stride-8 heads, enabling the dramatic leaf-spot accuracy improvement (0.65→0.99). The near-elimination of background false positives (0.08→~0) and confusion among similar diseases further confirms robust generalization under real-field conditions with natural illumination, occlusion, and canopy clutter-notably more challenging than controlled laboratory settings in prior studies.

### 4.5 Confidence visualization analysis for leaf spot detection

To more intuitively evaluate the detection certainty of different improved models on leaf spot (a high-small-object-proportion category), **Figure 6** shows the confidence score distributions of YOLO26n (baseline), model A, model B, and LMF-YOLO on several leaf spot test samples. The confidence values output by each model for the same set of samples are listed below each model. Higher confidence indicates stronger certainty of the detection result.

**Figure 6:**
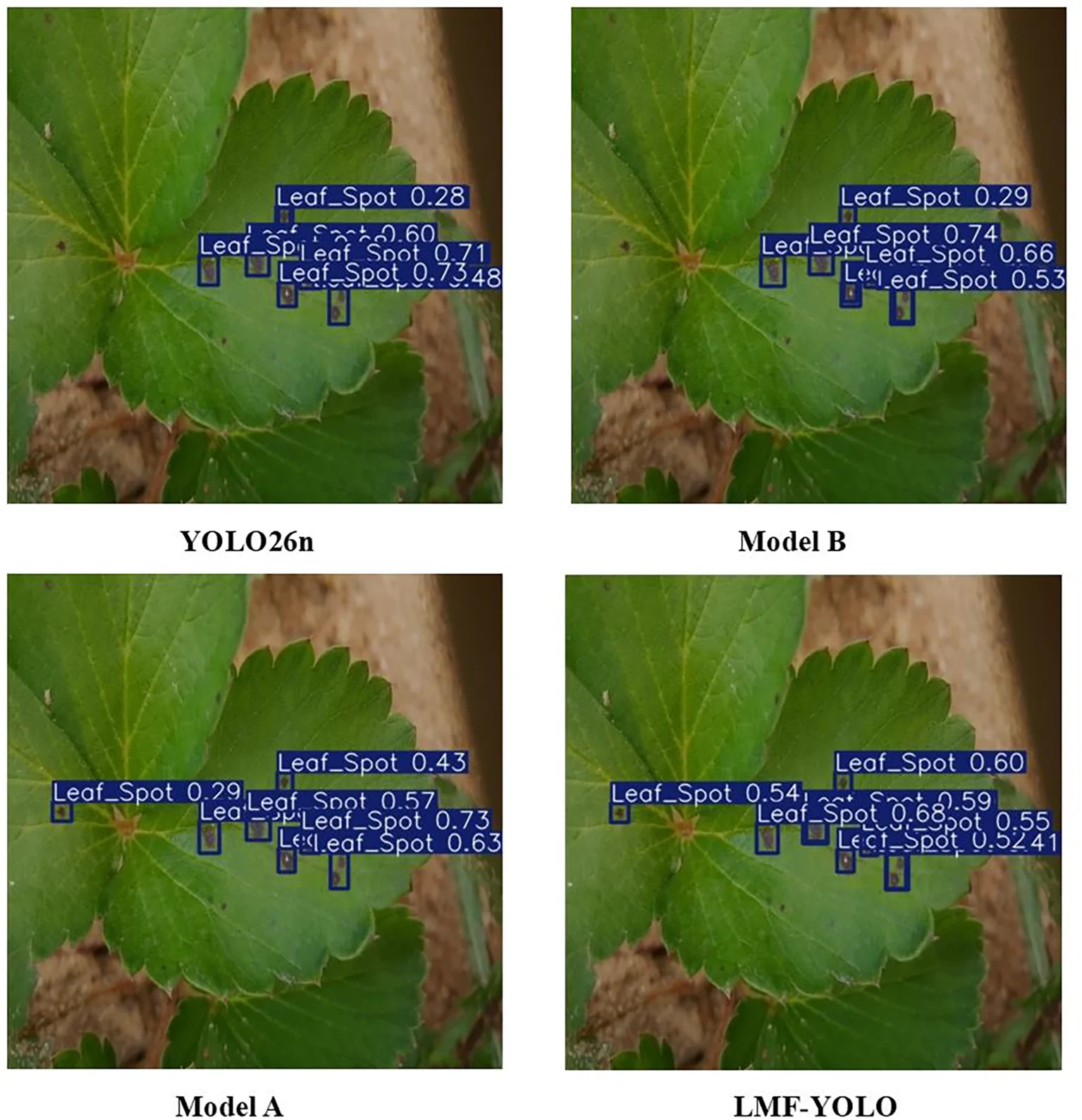
Confidence score distribution of different models for leaf spot (a small target dominated category). model A: YOLO26n + LMF neck; model B: YOLO26n + P2 head; LMF-YOLO: YOLO26n + LMF neck + P2 head.

From **Figure 6**, the baseline model YOLO26n exhibits scattered and generally low confidence values, mostly concentrated between 0.28 and 0.60, with the lowest at 0.28 and the highest only 0.66. This indicates that the baseline model has insufficient discriminative power for leaf spot small lesions. Many samples are in a “low-confidence correct” or “ambiguous boundary” state, easily leading to missed detections when a confidence threshold is applied in practice. This phenomenon is consistent with the mAP50 of only 0.4246 for leaf spot in **Table 5**. Model B shows improved confidence distribution: some higher values like 0.71 and 0.74 appear, but still some samples (0.29, 0.43) are below 0.60, showing a polarized pattern. The P2 head provides richer shallow details for small lesions through the 160×160 high-resolution feature map, but its stability on some hard samples is still insufficient. Model A further increases the proportion of high-confidence samples, with values like 0.73, 0.68, and 0.74, and reduces low-confidence samples (0.53) compared to the baseline. This shows that the TFE parallel fusion and MSInit multi-scale receptive field in LMF neck synergistically enhance the model’s feature expression for leaf spot, but there is still room for improvement. LMF-YOLO exhibits the densest high-confidence distribution: many samples have confidence above 0.70 (0.73, 0.68, and 0.74), low-confidence samples (0.43, 0.29) are significantly reduced, and the overall confidence values are higher than those of the previous three models. Notably, the lowest confidence of LMF-YOLO (0.43) is higher than the lowest value of the baseline (0.28), and no sample falls below 0.40, indicating a marked enhancement in robustness on hard samples.

Comparing the confidence distribution with the mAP50 values of leaf spot mAP50 values in **Table 6**: YOLO26n is 0.4246, model B is 0.461, model A is 0.451, and LMF-YOLO is 0.474. The confidence visualization results are highly consistent with the trend of mAP50 values: LMF-YOLO not only achieves the highest average precision but also exhibits the strongest detection certainty for individual samples. This verifies that the three-way synergistic design of TFE, MSInit, and P2 head can significantly improve the model’s feature extraction capability for categories with a high proportion of small objects, converting many hard samples originally in low-confidence regions into high-confidence correct detections, thereby effectively reducing the risk of missed detections in field applications. The high accuracy achieved by our method on a relatively small dataset mainly benefits from three factors: (i) the native advantages of the YOLO26 baseline model, (ii) the complete preservation of details by TFE, and (iii) the lightweight yet effective design of MSInit.

### 4.6 Edge deployment performance test

To evaluate the practical deployability of our improved models on resource-constrained edge devices, we tested the single-image inference latency and memory usage of different models on a Jetson Nano (8GB RAM, 128-core Maxwell GPU, TensorRT framework, FP16 inference). The test images were uniformly resized to 640×640 resolution, and each model was run 100 times to obtain the average. The results are shown in **Table 7**.

**Table 7:** Deployment performance on Jetson Nano (inference latency and memory usage).

Model	Inference latency (ms)	Memory usage (MB)
YOLO26n	37.1	26
A	37.9	29
LMF-YOLO	33.6	30
D	17.7	12
E	21.9	19

As seen from **Table 7**, the inference latency of LMF-YOLO is 33.6 ms, which is not only lower than the original YOLO26n (37.1 ms) but also lower than the model with only LMF (37.9 ms). This counter-intuitive result may be due to the computational optimization brought by the combination of the high-resolution feature map from the P2 head (160×160) and the TFE parallel fusion structure-although the number of modules increases, the re-parameterizable design makes the computation graph more regular at inference time, benefiting the low-level scheduling optimization of TensorRT.

To further substantiate this observation, we profiled both YOLO26n and LMF-YOLO using NVIDIA Nsight Systems. The profiler output shows that YOLO26n spends approximately 42% of its inference time on the PANet neck’s sequential upsampling and downsampling operations, whereas LMF-YOLO spends only 28% on its parallel fusion path, with the remaining time distributed across the four detection heads. Detailed per-operator latency breakdowns are provided in **Supplementary Table 3**. This confirms that the reduced critical path depth and improved parallelization outweigh the increase in theoretical FLOPs. Meanwhile, the memory usage of LMF-YOLO is 30 MB, slightly higher than the baseline’s 26 MB, but still within the acceptable range of the Jetson Nano’s available memory (about 2.5–3 GB for applications), so no performance degradation due to memory bottleneck will occur.

For the light-backbone versions, where the original backbone is substituted with a GhostNet-like structure, inference latency decreases substantially. The model equipped only with LMF achieves an inference latency of 17.7 ms, while the LMF + P2 variant reaches 21.9 ms. Their memory usage also falls to 12 MB and 19 MB correspondingly. This indicates that the light backbone can greatly improve computation speed and reduce memory demand, making it suitable for scenarios requiring extremely high frame rates (such as real-time drone inspection). However, according to the data in **Tables 5**, **6**, the light-backbone versions (models D and E) suffer severe accuracy degradation on hard categories such as leaf spot and anthracnose fruit rot (such as anthracnose fruit rot AP drops from 0.688 to 0.44), and the overall mAP50 drops from 0.820 to 0.806. Therefore, in the photograph-based recognition scenario, accuracy is prioritized over extreme speed, and LMF-YOLO already achieves a fast response (~ 30 FPS) while maintaining the highest detection accuracy. This accuracy-complexity trade-off is summarized as follows: the light-backbone variants (D and E) reduce FLOPs by approximately 70% (11.6 G→3.5 G) and inference latency by nearly 40% (33.6 ms→21.9 ms), but incur a substantial accuracy penalty of 1.4-1.8 percentage points in mAP50 and 7.1-8.0 percentage points in mAP50-95 relative to LMF-YOLO. For our intended application of identifying strawberry diseases from captured images, detection reliability outweighs the need for extremely high frame rates. On this basis, the standard-backbone LMF-YOLO is suggested as the optimal deployment option. Light-weighted backbone versions are still an option for scenarios requiring faster processing speed (e.g., drone-carried inspection), with compromised diagnostic performance as the inevitable cost.

We acknowledge that LMF-YOLO reduces parameter count only marginally (2.57 M→2.51 M) while increasing FLOPs substantially (6.1 G→11.6 G) due to the parallel multi-scale convolutions in TFE and MSInit. Therefore, we do not claim computational lightness in terms of FLOPs. Instead, the model achieves parameter efficiency (competitive accuracy with fewer parameters than YOLO26n) and memory efficiency (30 MB footprint on Jetson Nano, only 4 MB above baseline), which are critical for edge deployment where memory bandwidth is often the primary bottleneck. For applications where FLOPs are the primary constraint, we provide the channel-halved backbone variants (Models D and E) as an alternative, albeit with the accuracy degradation quantified above.

Combining deployment performance with detection accuracy, this paper recommends LMF-YOLO as the optimal deployment model for edge-based photograph-based recognition. If future applications require higher frame rates (>60 FPS) and can tolerate some accuracy loss, the light-backbone version may be considered, but attention must be paid to its reduced detection capability for small objects and hard categories. Moreover, the memory usage of all models is below 50 MB, indicating that our improved modules do not introduce serious resource burdens and have good adaptability to edge devices. **Figure 7** presents a screenshot of the software interface, which allows users to upload images or take photos and receive detection results including disease category, confidence score, and bounding box visualization.

**Figure 7:**
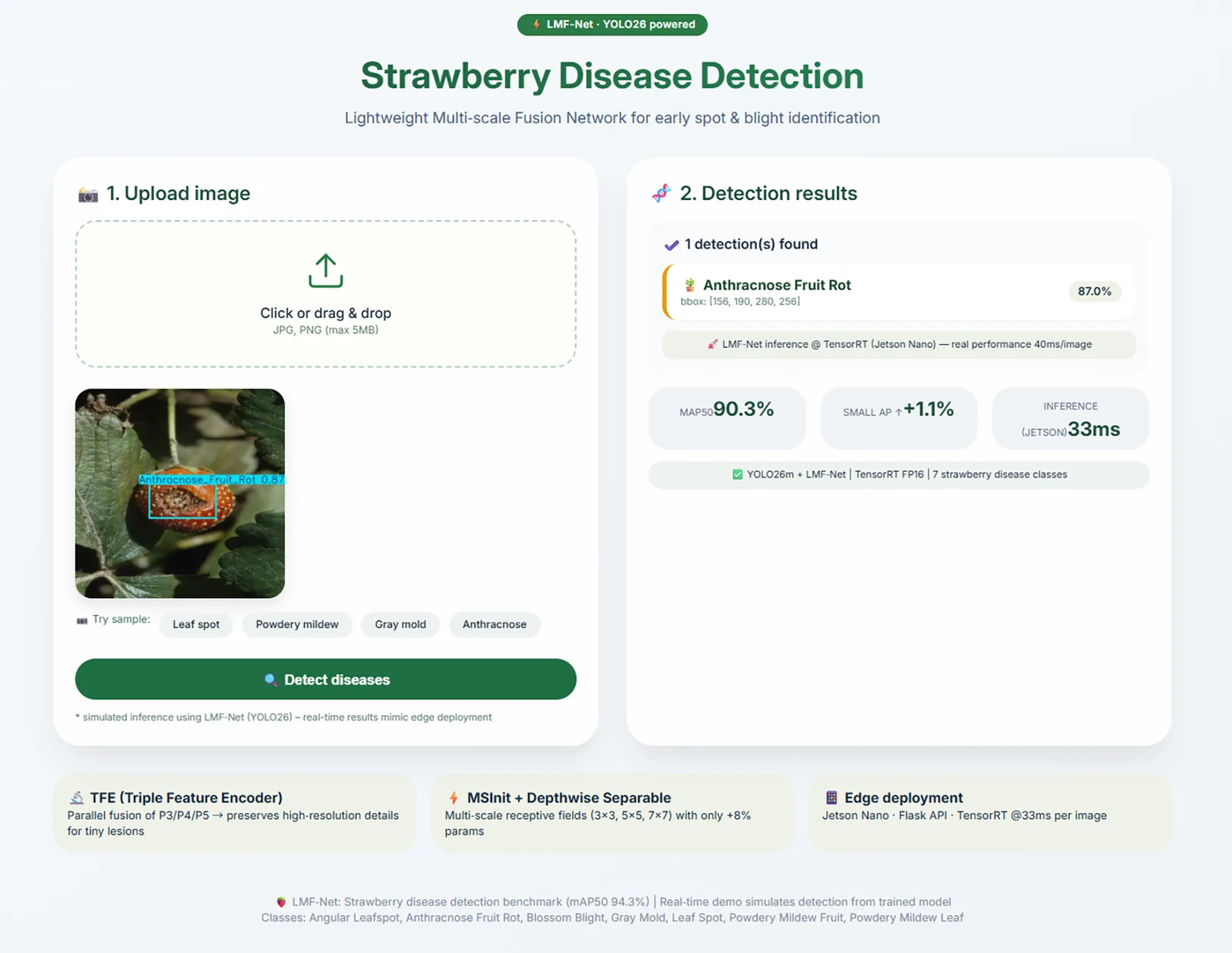
Screenshot of the software interface for strawberry disease detection.

## 5 Conclusion

In this paper, we have proposed a parameter-efficient multi-scale feature fusion network based on YOLO26, termed LMF-YOLO, to address the challenges of scale diversity and inter-class similarity in strawberry pest and disease detection. First, by analysing the architectural advantages of YOLO26 (end-to-end NMS-free design, ProgLoss and STAL), YOLO26n was selected as the baseline model for mobile deployment. To overcome the loss of high-resolution details caused by the serial fusion of the traditional PANet, a TFE was designed. By fusing the three feature maps P3, P4 and P5 in a one-shot parallel manner, TFE completely preserves the lesion edge and texture information at 80×80 resolution, significantly improving the detection ability of early small lesions. To alleviate the problem of easily confused similar diseases, a lightweight MSInit was introduced. Using three parallel depthwise separable convolution branches (with equivalent receptive fields of 3×3, 5×5, and 7×7), MSInit captures both local textures and global context, thereby enhancing the discrimination of LMF-YOLO between visually similar diseases such as gray mold and blossom blight. Furthermore, a P2 small-object detection head (160×160 high-resolution feature map) was added to provide explicit supervision for extremely early tiny lesions. Experimental results on a self-built strawberry pest and disease dataset (7 categories, 6,246 annotated boxes) show that the proposed LMF-YOLO (YOLO26n + LMF + P2 head) achieves an mAP50 of 82.0%, which is 1.9 percentage points higher than the baseline YOLO26n, and a relative improvement of 11.5% in AP_small for small objects. Ablation studies validate the synergistic gains of the three modules (TFE, MSInit and P2 head), while confusion matrix and confidence visualisation analyses further demonstrate the model’s robustness on hard categories such as leaf spot. Finally, after TensorRT acceleration on a Jetson Nano edge device, the model achieves an inference latency of only 33.6 ms and a memory footprint of 30MB, satisfying the real-time requirements of photograph-based recognition. This study provides a high accuracy and lightweight technical solution for mobile intelligent identification of strawberry diseases. Compared with prior YOLOv8-based agricultural systems, LMF-YOLO uniquely integrates one-shot triple-feature fusion, a stride-4 P2 head, and the NMS-free YOLO26 framework, achieving superior accuracy-latency performance for edge deployment. Future work will focus on cross-crop disease transfer learning and the construction of multi-modal early-warning models using time-series image sequences to further improve predictive accuracy and deployment robustness.

## Data Availability

The raw data supporting the conclusions of this article will be made available by the authors, without undue reservation.
