# Supplementary material for: LMF-YOLO: a multi-scale inception model for edge-based strawberry disease detection
Source: Front Plant Sci. 2026 Sep 30;17:1884996. doi: 10.3389/fpls.2026.1884996 (PMC13662136; doi:10.3389/fpls.2026.1884996)
Supplement: Supplementary file 1 [file Table1.docx]

**LMF-YOLO: A Multi‑scale Inception Model for Edge‑based Strawberry Disease Detection**

Yuzhen Niu, Ping Lin^*^

College of Chemical Engineering and Environment, Weifang University of Science and Technology, Weifang, 262700, China

E-mail:

Correspondence: linping@wfust.edu.cn

Table S1. Per‑operator latency breakdown on Jetson Nano.

| Model | Neck/Backbone | Detection Heads | Pre/Post‑processing | Total (ms) |
| --- | --- | --- | --- | --- |
| YOLO26n | 15.6 (42%) | 18.9 (51%) | 2.6 (7%) | 37.1 |
| LMF‑YOLO | 9.4 (28%) | 21.2 (63%) | 3.0 (9%) | 33.6 |

Table S2. Ablation study on TFE upsampling strategies.

| Upsampling Strategy | mAP50 | AP_small | Params (M) |
| --- | --- | --- | --- |
| Nearest‑neighbor（ours） | 0.820 | 0.107 | 2.51 |
| Bilinear | 0.817 | 0.103 | 2.51 |
| Transposed convolution | 0.818 | 0.104 | 2.57 |

Table S3. Sensitivity analysis on MSInit kernel size combinations.

| Kernel Size Combination | mAP50 | mAP50‑95 |
| --- | --- | --- |
| [3, 5, 7]（ours） | **0.820** | **0.640** |
| [3, 5, 5] | 0.816 | 0.637 |
| [3, 3, 7] | 0.814 | 0.635 |
| [5, 5, 7] | 0.812 | 0.633 |
| [1, 3, 5] | 0.809 | 0.630 |
